# A Stroke of Luck: Central Diabetes Insipidus Unmasked by a Heat Stroke

**DOI:** 10.7759/cureus.30768

**Published:** 2022-10-27

**Authors:** Abhinav Karan, Hui Jun Guo, Aaron Winer, Mike Ghobrial, Radhika Sharma, Pramod Reddy

**Affiliations:** 1 Internal Medicine, University of Florida College of Medicine – Jacksonville, Jacksonville, USA; 2 Internal Medicine, University of Florida College of Medicine, Gainesville, USA

**Keywords:** anti-diuretic hormone, hyperglycemia, hypernatremia, heat stroke encephalopathy, hyperthermia crisis, diabetes insipidus, diabetes

## Abstract

Diabetes insipidus is a rarely encountered cause of hypernatremia, often presenting a diagnostic and therapeutic dilemma for the encountering physician. Patients are often asymptomatic for a number of years due to compensation of their polyuria with polydipsia, but may have dramatic presentations in situations where they lose access to hydration. Our case is of a 62-year-old woman who was found unconscious with signs and symptoms of a heat stroke, and later was found to have resistant hypernatremia that persisted despite extensive free water supplementation. She had dilute polyuria throughout her hospital course, eventually warranting testing for diabetes insipidus with a vasopressin challenge test. She responded well to therapy with intranasal desmopressin and currently remains asymptomatic. Because our patient was reported to have polyuria and polydipsia for a number of years presumed to be due to underlying diabetes mellitus, it is possible that she had pre-existing central diabetes insipidus that was exacerbated by the lack of access to free water while she was in her intubated and sedated state. Alternatively, she may have also developed new-onset diabetes insipidus due to severe hyperthermia. This case serves to highlight a dramatic presentation of diabetes insipidus, and the importance of careful consideration of its diagnosis in patients with persistent dilute polyuria despite signs of intravascular volume depletion.

## Introduction

Diabetes insipidus is a rarely encountered disease that is challenging to diagnose in clinical practice. Many patients are relatively well for a number of years, with their only symptom being polydipsia. In our case, we present a patient who had polydipsia for many years that was misattributed to her diabetes mellitus. When she was admitted after a heat stroke and found to have severe hypernatremia unresponsive free water repletion, she was diagnosed with diabetes insipidus.

## Case presentation

A 62-year-old female, with a past medical history of poorly controlled type 2 diabetes mellitus and prior ischemic stroke one year prior, presented after being found unconscious at the side of her swimming pool. She was last seen in her normal state of health four hours prior to her discovery. She was hyperthermic with a temperature of 108°F on the arrival of emergency medical services and required emergent intubation for airway protection. On arrival to the emergency department, her initial laboratory testing revealed hyperglycemia to 440 mg/dl, and a corrected sodium concentration of 151 mmol/L (reference range: 135-145mmol/L). Her serum osmolality was initially elevated to 383mosm/kg [reference range: 274-298mosm/kg]. After active internal cooling and rehydration with cooled intravenous lactated ringer's and external ice pack application, she became normothermic and was successfully extubated. Her glucose improved to 189 mg/dl with insulin. However, her altered mentation persisted, and on repeat laboratory evaluation, a significant elevation of her sodium to 175 mmol/L was noted. She was initially presumed to be dehydrated due to her initial presentation and free water replacement was initiated with D5W and free water flushes through a nasogastric tube. Her sodium remained persistently elevated despite free water replacement, and she was noted to be polyuric throughout her admission, frequently with more than 3-4 L of documented urine output daily. Her urine osmolality was measured and found to be 104 mosm/kg (reference range: 300-900 mosm/kg), and total urine output over 24 hours was elevated at 5.2L (Table 1). 

**Table 1:** Reference values for urine output, sodium concentration, and urine osmolality before and after vasopressin challenge test

	On admission	Following Vasopressin Challenge
Urine Output (L/d)	5.2	1.4
Sodium (mmol/L)	175	143
Urine Osmolality (mosm/kg)	104	453

**Figure 1:**
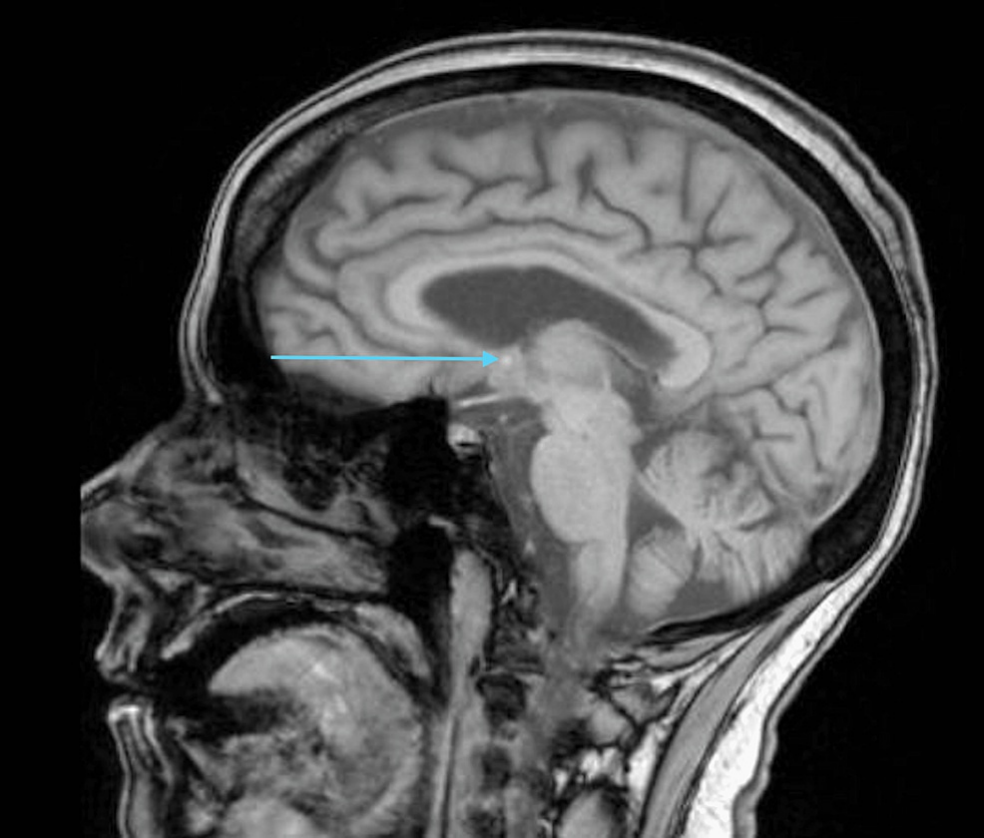
Sagittal T1 MRI brain and pituitary gland with hyperintense signal concerning for an ectopic posterior pituitary gland Blue arrow: Hyperintense signal concerning for posterior pituitary gland

On retrospective history taking, she denied any past medical history of malignancy, infiltrative disease, brain surgery, or other cranial trauma. Her family reported that they noticed significant polydipsia over the past year, alongside frequent visits to the bathroom, which they believed was secondary to diabetes mellitus. A vasopressin challenge test was subsequently conducted, resulting in a reduction in urine output to 1.4 in 24hrs, with a significantly increased urine osmolality to 453 mosm/kg (Table 1). A diagnosis of central diabetes insipidus was confirmed and subsequent MRI of the brain and the pituitary was concerning for an ectopic posterior pituitary gland (Figure 1). Following this, intranasal desmopressin was initiated with her sodium levels improving to 143 mmol/L by the end of her hospital stay. In addition, her mentation returned to baseline. She was discharged and has been doing well on follow-up and has remained compliant on her intranasal desmopressin.

## Discussion

Diabetes insipidus is rare, seen in only 1 in 25,000 individuals [1]. The diagnosis is often under-recognized, as the vast majority of patients will compensate with polydipsia, without other apparent clinical symptoms. Co-existing diabetes mellitus makes diabetes insipidus challenging to diagnose due to the shared symptomatology of polydipsia and polyuria. Significant polyuria despite intravascular volume depletion, as well as persistent polyuria despite glycemic control, can assist in differentiating between diabetes insipidus and diabetes mellitus in clinical practice. Furthermore, urine osmolality is a useful parameter that should be assessed in patients with polyuria and polydipsia.

Our patient’s worsening hypernatremia was initially attributed to water wasting and the inability to adequately self-hydrate due to her intubated and sedated state. However, after free water repletion, the patient continued to complain of persistent dilute polyuria throughout her hospitalization, despite supplemental free water replacement. This raised clinical suspicion for other infrequently encountered causes of hypernatremia such as central diabetes insipidus. Clinicians should consider challenging such patients with desmopressin to evaluate for the improvement of their polyuria, particularly in patients producing a large quantity of dilute urine when intravascular volume depletion is suspected.

The etiology of our patient’s diabetes insipidus also warrants discussion. It is plausible that our patient had partial diabetes insipidus due to a history of ischemic stroke occurring one year prior to her current presentation. One could also postulate that the heat stroke may have caused neuronal damage to areas of the pituitary and either resulted in new-onset diabetes insipidus or exacerbated pre-existing diabetes insipidus [2]. An ectopic posterior pituitary is normally reported as part of a congenital disease associated with pituitary stalk interruption syndrome or septo-optic dysplasia, reported in the literature as an etiology of diabetes insipidus in children [3]. It is otherwise unclear what significance this finding on imaging has for adult patients, and more research is warranted.

Overall, diabetes insipidus remains a challenging entity to diagnose and a high clinical suspicion should be warranted in patients with significant polyuria despite signs of intravascular volume depletion.

## Conclusions

Diabetes insipidus remains a challenging diagnosis in clinical practice, and is infrequently considered as a cause of hypernatremia due to its insidious presentation. Its possibility however, should be considered in all patients presenting with persistent dilute polyuria despite signs of intravascular volume depletion, and persistent hypernatremia unresponsive to free water repletion. Clinicians should also be aware of the possibility of a confounding presentation, particularly in patients with pre-existing diabetes mellitus with polyuria and polydipsia. 
